# LncRNA CKMT2-AS1 Promotes Hepatocellular Carcinoma Development Via Sponging miR-142-5p and Targeting IFITM3

**DOI:** 10.5152/tjg.2025.24547

**Published:** 2025-07-16

**Authors:** Xiaobo Ding, Chengming Jiao, Yitian Zou, Zheng Han, Shengjin Liu

**Affiliations:** 1 Department of Hepatobiliary, Jiangyin Liver and Gaiibiadder Hospital of Traditional Chinese Medicine, Jiangyin, China; 2 Department of Administrative, Jiangyin Liver and Gaiibiadder Hospital of Traditional Chinese Medicine, Jiangyin, China; 3 Nanjing University of Chinese Medicine, Nanjing, China

**Keywords:** Hepatocellular carcinoma, IFITM3, LncRNA, miRNA

## Abstract

**Background/Aims:**

Hepatocellular carcinoma (HCC) stands as the foremost contributor to cancer-related mortality, underscoring its profound significance in the oncological landscape. This study explores the potential regulatory mechanisms of lncRNA CKMT2-AS1 in the functionality of HCC cells, thereby providing a basis for therapeutic approaches in the treatment of HCC.

**Materials and Methods:**

Quantitative polymerase chain reaction (qPCR) was utilized to evaluate the expression of CKMT2-AS1 and miR-142-5p in HCC tissues and cells. The interactions between CKMT2-AS1 and miR-142-5p, as well as the interplay between IFITM3 and miR-142-5p, were confirmed through dual-luciferase assays. To assess the effects of CKMT2-AS1 transfection, alongside the co-transfection of CKMT2-AS1 and miR-142-5p interference plasmids, a series of experiments were conducted utilizing CCK-8 and transwell assays to evaluate changes in proliferation, migration, and invasion of HCC cells. Additionally, qPCR was employed to elucidate the influence of miR-142-5p on the expression of IFITM3 in HCC cells.

**Results:**

The study elucidated that CKMT2-AS1 was significantly upregulated in HCC tissues and cells, while miR-142-5p exhibited a marked downregulation, revealing a noteworthy negative correlation. Notably, the downregulation of CKMT2-AS1 hindered the function of HCC cells. Conversely, the downregulation of miR-142-5p effectively mitigated the inhibitory effects of CKMT2-AS1 on cellular activities. Furthermore, the upregulation of miR-142-5p resulted in a substantial decrease in the levels of IFITM3 in HCC cells. This investigation established a potential regulatory network, identifying IFITM3 as the downstream target mRNA.

**Conclusion:**

The sponging effect of CKMT2-AS1 on miR-142-5p resulted in altered expression levels of IFITM3, subsequently influencing the progression of HCC.

**Main Points**

- CKMT2-AS1 shows markedly upregulated in hepatocellular carcinoma (HCC) tissues and cells.
- CKMT2-AS1 and miR-142-5p have a notable negative correlation.
- CKMT2-AS1 enhances HCC cell proliferation, migration, and invasion.
- CKMT2-AS1 promotes HCC development via sponging miR-142-5p and targeting IFITM3.

## Introduction

Hepatocellular carcinoma (HCC) ranks as the sixth most prevalent malignant neoplasm worldwide, with a disheartening 5-year survival rate of less than 9%.^1^ Currently, the primary therapeutic approaches for HCC include surgical resection, liver transplantation, and ablation therapy.^2^ The insidious nature of early symptoms and the challenges associated with accurate early diagnosis frequently result in patients being diagnosed at advanced stages of the disease. This reality often forecloses the possibility of optimal surgical intervention, necessitating the reliance on conservative treatment, and alternatives such as chemotherapy, immunotherapy, and targeted drug therapy.^3^ Unfortunately, individual variability significantly impacts treatment tolerability; more than half of the patients experience intolerance to these therapeutic strategies, exacerbated by a high prevalence of adverse reactions, that adversely affect prognosis and substantially reduce patient survival rates.^4^ The pathogenesis and treatment of HCC continue to be critical domains of clinical research. Consequently, an enhanced understanding of the pivotal molecular targets and potential mechanisms underlying liver cancer may pave the way for the development of more effective therapeutic strategies for those afflicted with HCC.

Long non-coding RNA (lncRNA) is conventionally characterized as RNA molecules with nucleotide sequences exceeding 200 bases that lack protein-coding capability. These molecules exhibit significant spatiotemporal and tissue-specific expression patterns across various tissues and organs.^5^ LncRNAs are increasingly acknowledged as crucial regulatory elements within gene expression networks.^6^ Numerous studies have elucidated the vital roles that lncRNAs play in regulating tumor cell functions.^7^ A recent investigation into tumors of the digestive tract revealed that several lncRNAs displayed differential expression patterns and demonstrated considerable prognostic significance. These findings offer novel avenues for the treatment and enhancement of associated diseases.^8^ LncRNA CKMT2-AS1, a recently identified lncRNA spanning 2,224 base pairs, has garnered attention for its close association with the initiation and progression of various tumors. In colorectal cancer (CRC), microarray analyses have revealed that CKMT2-AS1 is downregulated in both CRC tissues and cell lines, implicating its involvement in autophagy processes. Furthermore, this lncRNA is capable of diminishing the survival of CRC cells by intricately modulating the AKT/mTOR signaling pathway.^9^ In the context of breast cancer, expression levels of CKMT2-AS1 are significantly reduced in tumor samples, indicating its potential as a biomarker for the disease.^10^ In papillary renal cell carcinoma (pRCC), CKMT2-AS1 exhibits notable upregulation in tumor tissues, emerging as a potential risk factor for the disease. A significant correlation has also been established between CKMT2-AS1 expression levels and disease severity, wherein patients exhibiting low expression levels are associated with a more favorable prognosis.^11^ Additionally, analyses from the Encyclopedia of RNA Interactomes (ENCORI) database indicate a marked upregulation of CKMT2-AS1 expression in HCC. However, investigations concerning CKMT2-AS1 in HCC remain sparse. Notably, it has been established that lncRNAs may function as molecular sponges for microRNAs (miRNAs) or splicing factors, thereby negatively regulating the expression of downstream target genes through interactions with specific miRNAs.^12^

miRNA represents a class of non-coding RNA molecules, typically comprising 18–24 nucleotides. Among these, miR-142-5p, located on human chromosome 7, plays a pivotal role as a tumor suppressor. Recent studies have revealed that miR-142-5p is significantly downregulated in HCC associated with hepatitis B virus (HBV) infection.^13^ Overexpression of miR-142-5p has been shown to inhibit cellular proliferation and promote apoptosis, thus contributing to the modulation of HCC progression.^14^ Additionally, miR-142-5p has been identified as a critical regulator of disease advancement by targeting essential signaling pathways, including MAPK, Wnt, VEGF, and Notch.^15^ Interferon-induced transmembrane protein 3 (IFITM3) acts as a surface marker for a diverse array of tumor stem cells, and research has demonstrated that the protein encoded by this gene is implicated in the progression of various malignancies.^16^ Notably, elevated levels of IFITM3 in liver cancer tissues can enhance cancer cell growth and proliferation, as well as increase their invasive and metastatic potential.^17^ However, the role and potential regulatory mechanisms of CKMT2-AS1 in HCC remain poorly understood, with a notable scarcity of reports examining the intricate regulatory network connecting lncRNA, miRNA, and mRNA in this context.

In light of the aforementioned research, this investigation centers on the differential expression of CKMT2-AS1 in HCC patients and its implications for liver cancer cell functionality. By delving into the targeted regulatory interactions among CKMT2-AS1, miR-142-5p, and IFITM3, the aim was to elucidate the molecular mechanisms through which CKMT2-AS1 influences the onset and progression of HCC. This study aspires to establish a novel theoretical framework for understanding the intricate molecular pathways underlying the development of this malignancy.

## Materials and Methods

### Study Participants and Tissue Collection

This study involved the selection of 66 patients diagnosed with HCC who received treatment at Jiangyin Liver and Gaiibiadder Hospital of Traditional Chinese Medicine from 2020 to 2023. The inclusion criteria for these HCC patients were as follows: (a) adherence to the diagnostic standards outlined in the Diagnosis and Treatment Guidelines for Primary Liver Cancer (2020 Edition); (b) the patient was undergoing surgical resection for the first time; (c) preoperative imaging confirmed the diagnosis of liver cancer, and subsequent pathological analysis of the resected specimen verified of HCC; (d) the patient had not received any form of radiotherapy, chemotherapy, targeted therapy, or related treatments prior to surgery; (e) the patient exhibited a stable mental state. Patients were excluded based on the following criteria: (a) individuals who had received preoperative radiotherapy, chemotherapy, targeted therapy, or any other related treatments; (b) those whose postoperative pathological diagnosis revealed a benign tumor or a concurrent malignant tumor at differing anatomical sites; (c) individuals with metastatic cancer resulting from the dissemination of tumors from other origins to the liver; (d) patients exhibiting severe dysfunction of the heart, liver, lungs, or kidneys prior to hospitalization; (e) pregnant or lactating individuals.

During surgical procedures, it is imperative to ensure that collected cancerous tissue samples are devoid of necrotic tissue. Additionally, non-tumor tissue specimens should be obtained from a location at least 3 cm distant from the tumor resection margin. The target sample size was approximately 1 cm^3^, which should be carefully placed in a 1.5 mL RNase-free tube. The entire sampling process was to be completed within a maximum time frame of 30 min, with all operations conducted under sterile conditions to maintain sample integrity. Following the collection of specimens, all samples would be promptly frozen in liquid nitrogen to ensure the preservation of their biological properties. It is important to note that all participants have provided informed consent, and the study protocol has received ethical approval from Jiangyin Liver and Gaiibiadder Hospital of Traditional Chinese Medicine (Date:February 1 ,2024/No.2024-LY-001).

### Cell Culture

Human liver cancer cell lines, namely Huh-7, HCCLM3, HepG2, and Hep3B, alongside the human normal liver cell line Immortalized Human Hepatocytes (MIHA/IHH), were procured from Shanghai Enzyme-Linked Biotechnology Co., Ltd. The complete dulbecco’s modified eagle medium (DMEM) culture medium was meticulously prepared by combining penicillin-streptomycin, fetal bovine serum (FBS), and DMEM basic medium (Sigma Aldrich, USA) in a precise ratio of 1:10:89. Similarly, the complete roswell park memorial institute (RPMI)-1640 medium was formulated utilizing RPMI-1640 basic medium (Sigma Aldrich, USA) in congruent proportions. For the minimum essential medium (MEM) complete medium, 10% FBS, 1% double reactor solution, 1% non-essential amino acids (NEAA, Gibco, USA), and 1 mM NaP (Cobioer, China) were incorporated into the MEM basic medium (Sigma Aldrich, USA). The procured cell suspension was evenly distributed into cell culture bottles, with 4 mL of DMEM complete culture medium added to the Huh-7 and HCCLM3 cells, 4 mL of MEM complete culture medium allocated to HepG2 and Hep3B cells, and 4 mL of RPMI-1640 complete culture medium provided for the MIHA cells. The cells were subsequently incubated in an incubator maintained at 5% CO_2_, a controlled temperature of 37°C, and a humidity level of 97%. Daily observations of cell growth were conducted. After achieving stable passage for 10 cycles, the cells were utilized for subsequent transfection experiments.

### Cell Transfection

As documented in prior research, small interfering RNA targeting CKMT2-AS1 (si-CKMT2-AS1) and its corresponding negative control (si-NC) were meticulously synthesized by Ribobio Biotech in Beijing, China. Additionally, the miR-142-5p inhibitor, along with its mimetic counterpart (miR-142-5p mimic) and their respective negative controls (inhibitor NC and mimic NC), were sourced from Shanghai GenePharma Pharmaceutical Technology Co., Ltd. The Huh-7 and HCCLM3 cell lines were cultured, and transfection was performed once the cells attained approximately 60% confluence. A total of 10 nM of the vectors were introduced into the cells utilizing Hieff Trans® Universal transfection reagent (Yeasen, China) for 24 hours.

### Nuclear-Cytoplasmic Separation

Cultivated the Huh-7 and HCCLM3 cell lines to the desired concentration. Following this, the cells were digested with 0.25% trypsin and transferred to RNase-free centrifuge tubes. The cell suspension was subjected to centrifugation at 3000 × g for 5 min to facilitate precipitation. The supernatant, which contained the culture medium, was discarded, and the cells were washed multiple times with phosphate buffered saline (PBS) to remove any remaining traces of the culture medium. Adhering meticulously to the protocol provided in the Paris kit (Ambion, USA) for the separation of cytoplasmic and nuclear components, RNA was extracted from both the cytoplasm and nucleus separately. Subsequently, qPCR was employed to evaluate the expression levels of CKMT2-AS1, utilizing U6 and *GAPDH* as internal reference genes.

### RNA Extraction and cDNA Synthesis

Total RNA was extracted utilizing the MolPure® The Cell/Tissue RNA Kit (Yeasen, China) and evaluated for quality following standard methodologies. A total of 1 μg of RNA served as the template for the synthesis of the first strand cDNA, employing the BeyRT™ III First Strand cDNA Synthesis Kit (Beyond, China). The resulting cDNA was subsequently stored at −20 °C for future applications. All procedures were meticulously carried out on ice, adhering strictly to established laboratory protocols and the detailed instructions provided with the reagent kits, thereby ensuring precision and minimizing the potential for errors throughout each phase of the process.

### Quantitative Polymerase Chain Reaction

The expression levels of CKMT2-AS1, miR-142-5p, and *IFITM3* were evaluated utilizing the BeyFast™ SYBR Green qPCR Mix (2X, High ROX) (Beyotime, China) and the HRbio™ miRNA qPCR gene expression detection kit (HeRui, China). The primer sequences for CKMT2-AS1, miR-142-5p, and *IFITM3* are detailed in Supplementary Table 1. Following the manufacturer’s protocol, a 20 μL qPCR reaction mixture was prepared. The detection was performed using the CFX Opus 96 system (Bio-Rad, USA), and the dissolution curve was generated according to the instrument’s default settings.

### Cell Proliferation Activity Assay

The transfected cells were subjected to digestion with trypsin and subsequently seeded into a 96-well plate, ensuring that 3 replicates were established for each experimental group. Once the cellular state had stabilized, cell proliferation was assessed utilizing the Cell Count-8 kit (CCK-8, Dojindo, Japan). A mixture of DMEM basic culture medium and the CCK-8 reagent was prepared in a 10:1 ratio and added to the 96-well plate. The plates were incubated in a dark environment for 2 h to facilitate the reaction. After incubation, the cells were collected, and the optical density (OD)_450 nm_ value was measured, with 3 readings taken for each well to ensure accuracy.

### Cell Migration and Invasion Assay

The concentration of transfected cells was adjusted in each experimental group to 5 × 10^4^ cells/mL utilizing DMEM basic medium. The cell suspension was then introduced into the upper insert of a Transwell chamber (Solarbio, China), followed by the addition of a complete culture medium into the lower insert. After a 48-hour incubation period, the cells were fixed with 4% paraformaldehyde and subsequently stained with 0.4% crystal violet. The cells were then observed and quantified using an inverted microscope. The procedure for the invasion assay closely mirrored that of the migration experiment; however, pre-cooled Matrigel matrix gel was diluted with DMEM basic medium at a ratio of 1:8 and applied to the Transwell chamber. After allowing the gel to dry naturally, 100 μL of the cell suspension was added. The subsequent steps were identical to those of the migration experiment.

### Luciferase Report Assay

The wild-type (WT) and mutant (MUT) sequences of CKMT2-AS1 and *IFITM3*, which include the binding sites for miR-142-5p, were cloned into the pmiR-GLO vector to construct the recombinant plasmids CKMT2-AS1-WT, CKMT2-AS1-MUT, IFITM3-WT, and IFITM3-MUT. Subsequently, these vectors were co-transfected into Huh-7 and HCCLM3 cells in the logarithmic growth phase using Lipofectamine 3000 transfection reagent, alongside miR-142-5p mimic and mimic NC. Luciferase activity in the cells was measured 48 hours post-transfection employing a dual luciferase reporter gene detection kit (Beyotime, China) and analyzed using a Luminoskan Ascent chemiluminescence reader (Thermo, USA).

### Data Analysis

This research employed GraphPad Prism 9.0 (GraphPad Software; San Diego, CA, USA) for comprehensive statistical analysis. The expression levels of CKMT2-AS1 and miR-142-5p across various tissues were assessed utilizing a T-test. Furthermore, Analysis of variance (ANOVA) was utilized to evaluate the differential expression of CKMT2-AS1, miR-142-5p, and *IFITM3* in various cell lines. Additionally, ANOVA was performed to analyze cell functional differences among distinct transfection groups. Pearson correlation analysis was utilized to elucidate the relationship between the expression levels of CKMT2-AS1 and miR-142-5p.

## Results

### Relative Expression Levels of lncRNA CKMT2-AS1

**Figure 1.**
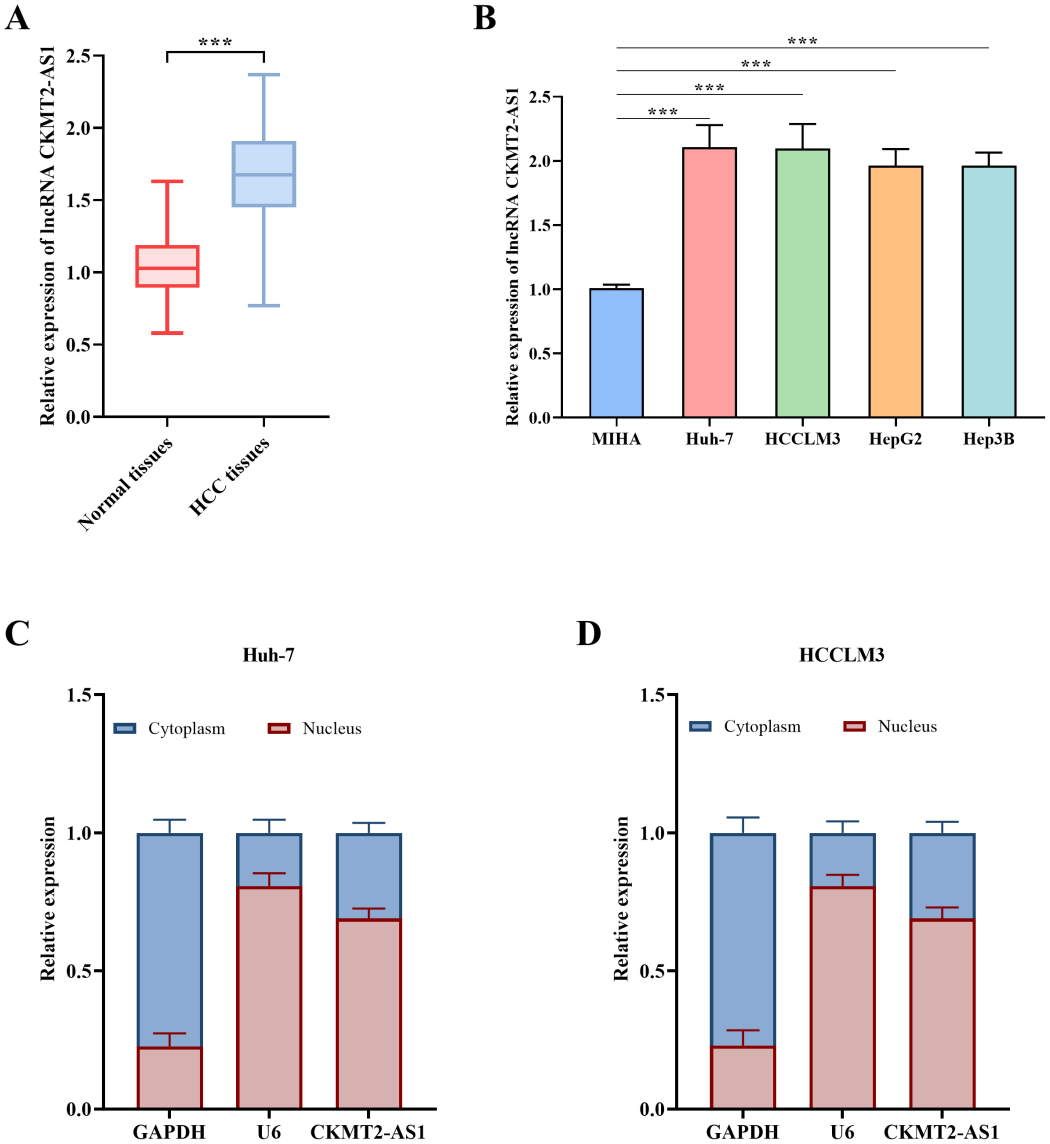
The relative expression levels of lncRNA CKMT2-AS1 in both normal and HCC tissues (A), as well as the levels observed in the normal liver cell line MIHA and liver cancer cell lines Huh-7, HCCLM3, HepG2, and Hep3B (B). The distribution of CKMT2-AS1 expression in the cytoplasmic and nuclear compartments of Huh-7 (C) and HCCLM3 (D) cells. ***, *P* < .001 vs. Normal tissues/MIHA.

The expression of CKMT2-AS1 was significantly elevated (*P* < .001, Figure 1A) in HCC tissues. Furthermore, when compared to MIHA cells, CKMT2-AS1 exhibited a marked increase in the Huh-7, HCCLM3, HepG2, and Hep3B cell lines (*P* < .001, Figure 1B). Notably, the highest expression levels of CKMT2-AS1 were observed in the Huh-7 and HCCLM3 cell lines, which were subsequently selected for further experimental investigation. The results obtained from the nuclear-cytoplasmic separation experiments revealed that CKMT2-AS1 was predominantly expressed within the nuclei of the Huh-7 and HCCLM3 cell lines (Figure 1C and D).

### Effect of Inhibiting CKMT2-AS1 on Hepatocellular Carcinoma Cells

**Figure 2.**
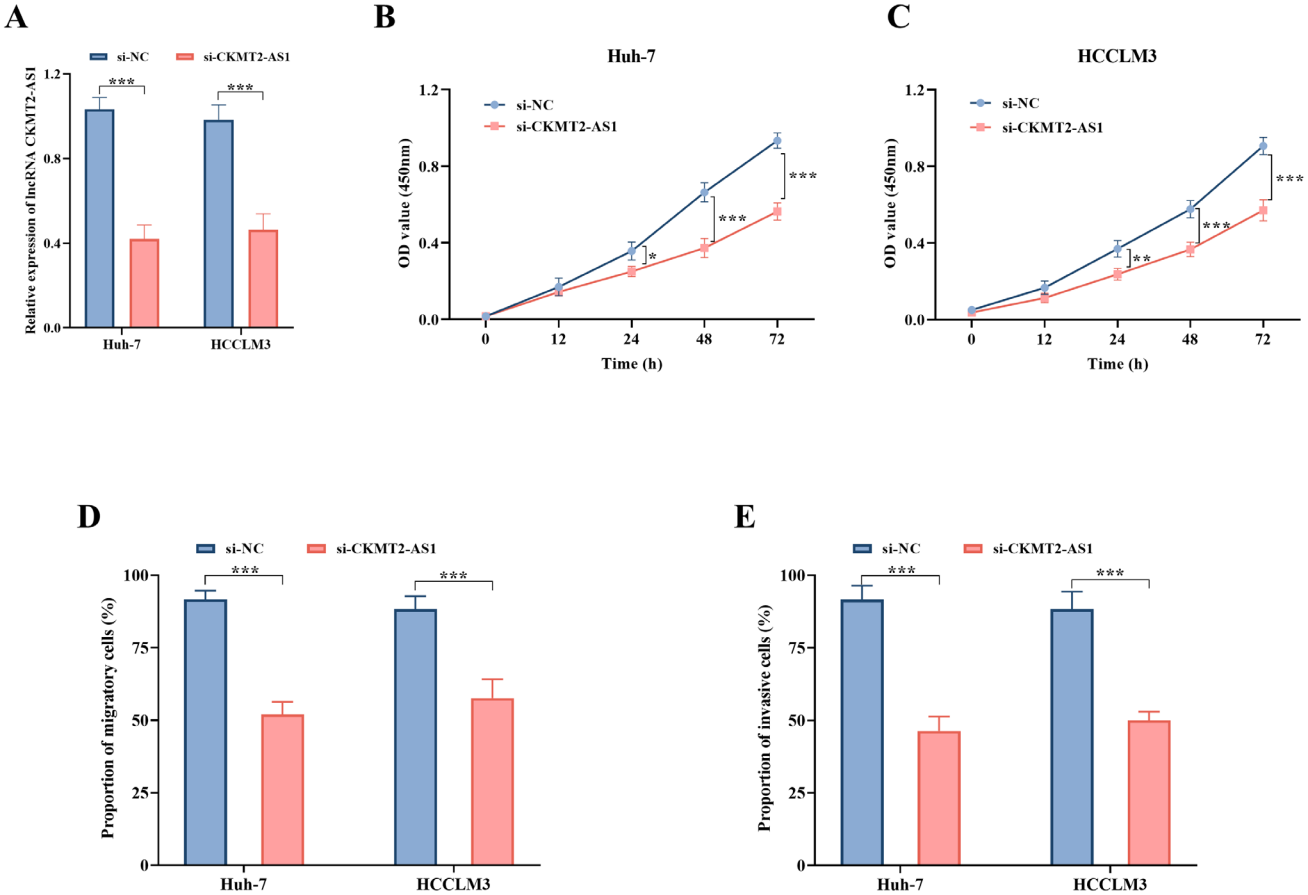
The relative expression levels of lncRNA CKMT2-AS1 (A), alongside the proliferation activity (B, C), migration (D), and invasion (E) abilities of Huh-7 and HCCLM3 cells in the si-NC and si-CKMT2-AS1 groups. *, *P* < .05; **, *P* < .01; ***, *P* < .001 vs. si-NC.

Upon the transfection of si-CKMT2-AS1 into Huh-7 and HCCLM3 cells, a noteworthy and statistically significant reduction in CKMT2-AS1 levels was observed (*P* < .001, Figure 2A). The down-regulation of CKMT2-AS1 for 24 h resulted in a marked inhibition of proliferative activity in both Huh-7 (*P* < .05, Figure 2B) and HCCLM3 (*P *< .01, Figure 2C) cells. Furthermore, the suppression of CKMT2-AS1 led to a substantial decrease in both the migratory (*P* < .001, Figure 2D) and invasive (*P* < .001, Figure 2E) capabilities of Huh-7 and HCCLM3 cells.

### Relationship Between CKMT2-AS1 and miR-142-5p

**Figure 3.**
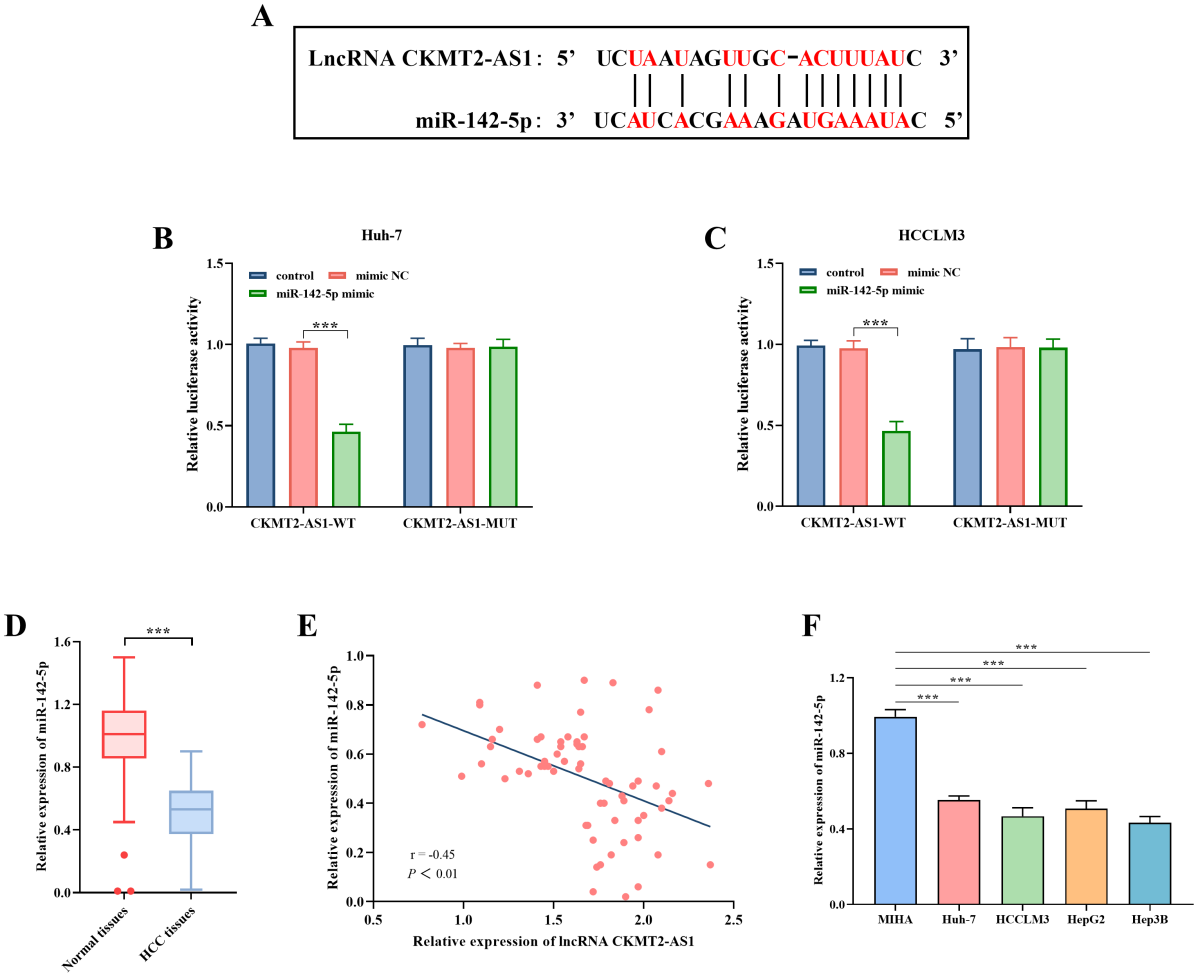
The binding sites between lncRNA CKMT2-AS1 and miR-142-5p (A), as well as the dual luciferase activity for both CKMT2-AS1-WT and CKMT2-AS1-MUT regulated by miR-142-5p in Huh-7 (B) and HCCLM3 (C) cells. The relative expression levels of miR-142-5p in normal and HCC tissues (D), and the correlation observed between lncRNA CKMT2-AS1 and miR-142-5p within HCC tissues (E), as well as the levels of miR-142-5p in normal liver cell line MIHA and various liver cancer cell lines, including Huh-7, HCCLM3, HepG2, and Hep3B (F). ***, *P* < .001 vs. mimic NC/Normal tissues/MIHA.

**Figure 4.**
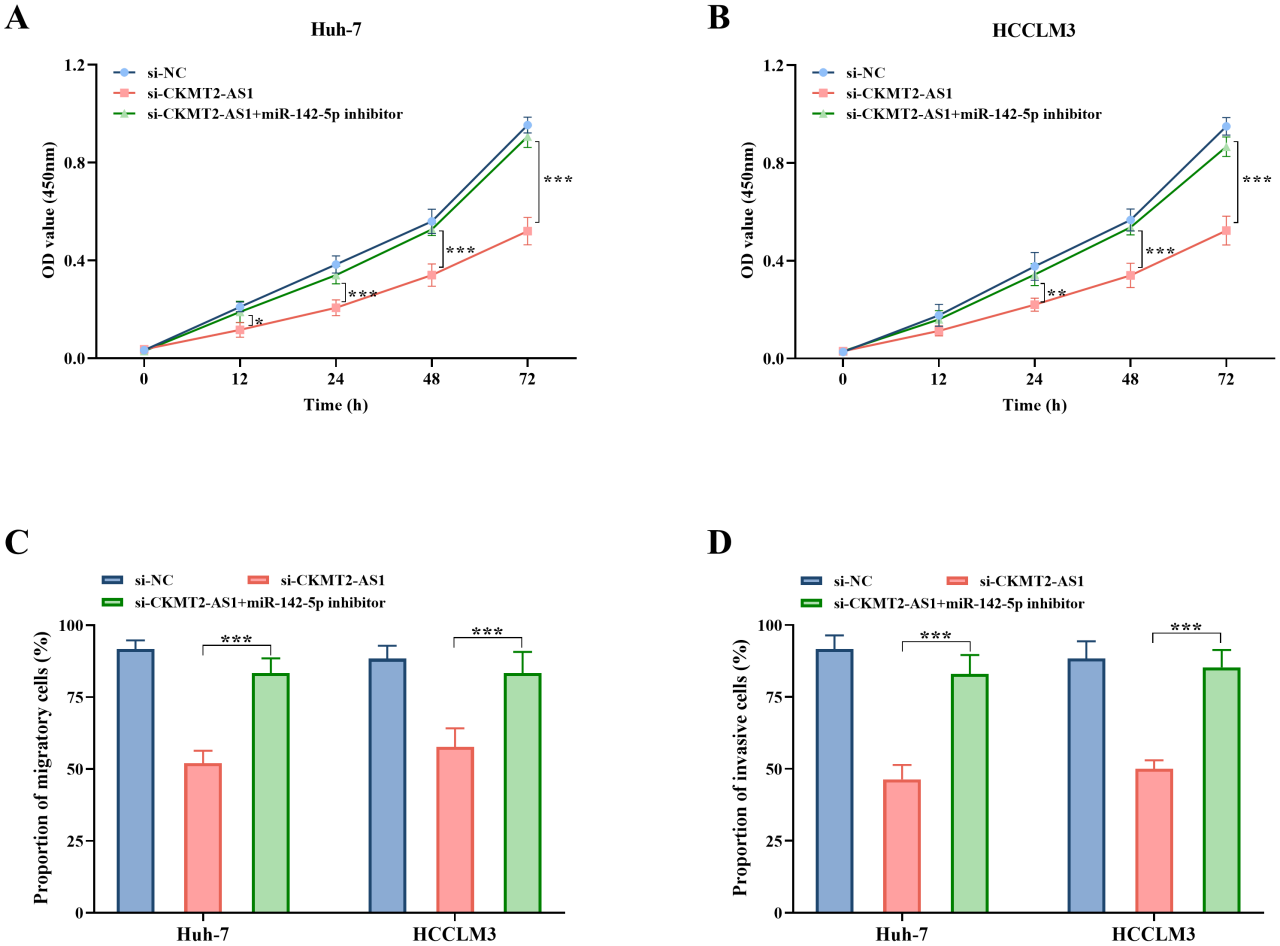
The proliferation activity (A, B), migration (C), and invasion (D) abilities of Huh-7 and HCCLM3 cells in the si-CKMT2-AS1 and si-CKMT2-AS1 + miR-142-5p inhibitor groups. *, *P* < .05; **, *P* < .01; ***, *P* < .001 vs. si-CKMT2-AS1.

The binding sites between CKMT2-AS1-WT and CKMT2-AS1-MUT with miR-142-5p were predicted utilizing the Encyclopedia of RNA Interactomes (ENCORI) database (Figure 3A). Subsequent experiments demonstrated that the introduction of miR-142-5p mimic resulted in a significant reduction in the luciferase activity of the CKMT2-AS1-WT vector in both Huh-7 and HCCLM3 cell lines (*P* < .001, Figure 3B and C). In contrast, no notable impact was observed on CKMT2-AS1-MUT. Notably, miR-142-5p expression was significantly decreased in HCC tissues (*P* < .001, Figure 3D). A significant negative correlation was identified between CKMT2-AS1 and miR-142-5p (r = −0.45, *P* < .01, Figure 3E). Additionally, miR-142-5p levels were significantly downregulated in the Huh-7, HCCLM3, HepG2, and Hep3B cell lines in comparison to MIHA (*P* < .001, Figure 3F). The results of co-transfection experiments indicated that the inhibition of proliferation due to CKMT2-AS1 silencing was significantly alleviated by the transfection of the miR-142-5p inhibitor (*P *< .05, Figure 4A and B). Furthermore, Transwell assays evaluating migration and invasion revealed that the inhibition of miR-142-5p could counteract the suppressive effects on migration (*P *< .001, Figure 4C) and invasion (*P* < .001, Figure 4D) associated with CKMT2-AS1 silencing.

### Relationship Between miR-142-5p and Interferon-Induced Transmembrane Protein 3

**Figure 5.**
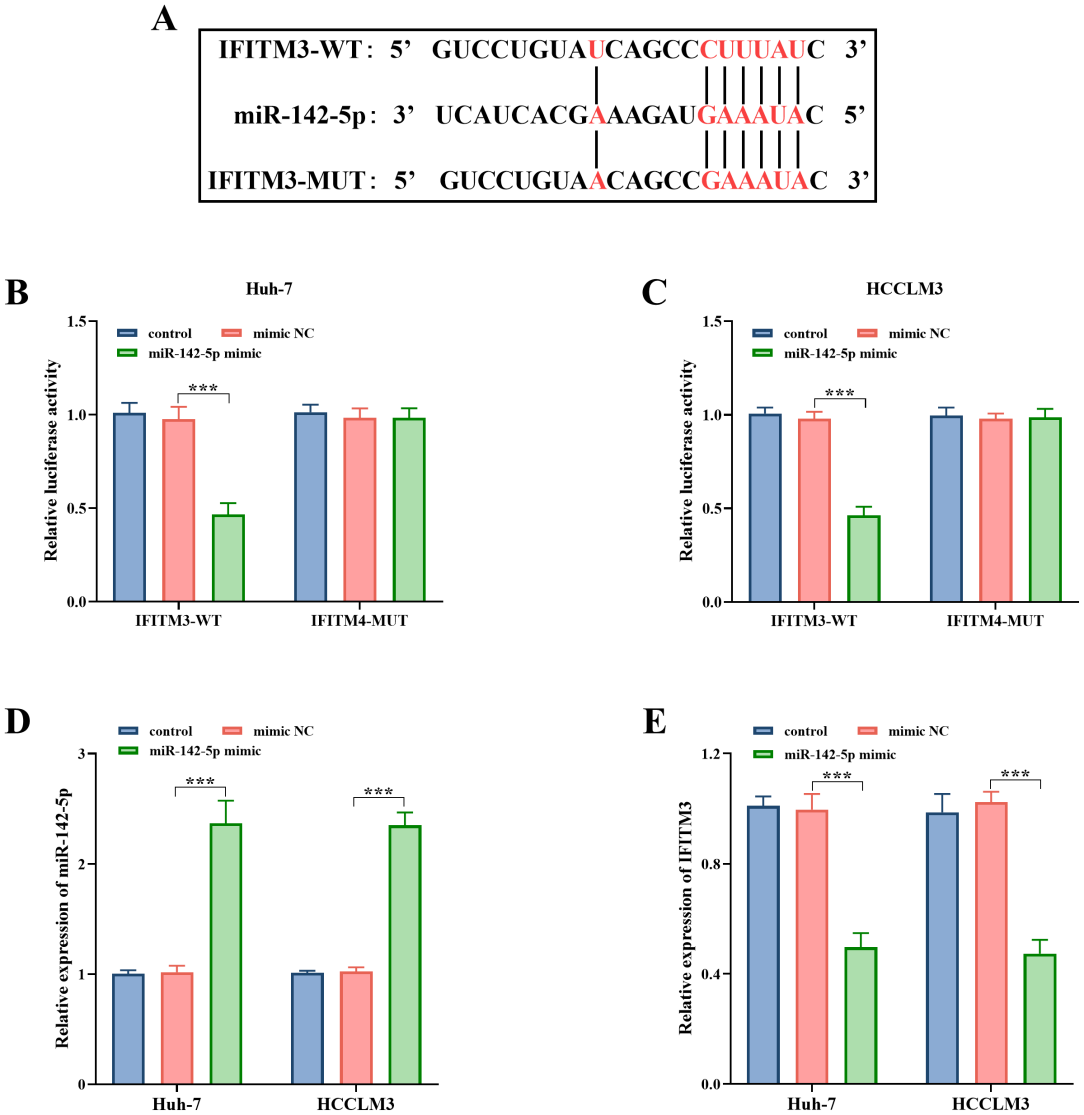
The binding sites of *IFITM3* and miR-142-5p (A), as well as the dual luciferase activity of IFITM3-WT and IFITM3-MUT as influenced by miR-142-5p in both Huh-7 (B) and HCCLM3 (C) cells. The relative expression levels of miR-142-5p (D) and *IFITM3* (E) in Huh-7 and HCCLM3 cells following treatment with the miR-142-5p mimic. ***, *P* < .001 vs. mimic NC.

The ENCORI analysis elucidated the interaction between miR-142-5p and the binding sites of *IFITM3* (Figure 5A). Subsequent experiments revealed that the overexpression of miR-142-5p led to a significant decrease in luciferase activity within the IFITM3-WT group (*P* < .001, Figure 5B and C). Furthermore, qPCR analysis indicated a significant increase in miR-142-5p levels (*P* < .001, Figure 5D), accompanied by a marked decrease in *IFITM3* expression (*P* < .001, Figure 5E) in HCC cells.

## Discussion

Despite significant advancements in the treatment of liver cancer, the prognosis for patients with HCC remains dismal, largely attributable to late-stage diagnoses, suboptimal efficacy of chemotherapy, and high recurrence rates.^18^ Numerous lncRNAs have been identified as exhibiting abnormal expression patterns in HCC, thereby influencing disease progression through the modulation of various cellular processes. For instance, lncRNA HULC ranks among the most prominently upregulated genes in HCC, promoting cell proliferation and metastasis by downregulating miR-372.^19^ Additionally, LINC01419 is significantly overexpressed in HCC tissues and in 4 distinct cell lines; its downregulation correlates with diminished functionality of HCC cells.^20^ As previously mentioned, CKMT2-AS1 demonstrates substantial downregulation in CRC^9^ and breast cancer tissues,^10^ where it plays a role in biological processes such as autophagy by modulating the activity of downstream target genes. Conversely, CKMT2-AS1 is markedly upregulated in pRCC, acting as a risk factor for poor prognosis.^11^ This observation suggests that CKMT2-AS1 may exert either oncogenic or tumor-suppressive effects depending on the context. Prior to conducting experimental studies, this investigation employed the ENCORI database, which predicted a notable upregulation of CKMT2-AS1 expression in HCC. Experimental findings confirmed a significant increase in CKMT2-AS1 levels in HCC tissues and in several cell lines (Huh-7, HCCLM3, HepG2, and Hep3B). Functional assays indicated that the inhibition of CKMT2-AS1 significantly impaired the functional capacity of HCC cells. Thus, CKMT2-AS1 appears to function as an oncogenic factor in HCC, warranting further exploration of its regulatory mechanisms at the cellular level.

Numerous studies have illuminated the role of lncRNAs as ceRNAs, thereby inhibiting the expression of downstream miRNAs and influencing tumorigenesis.^21^ In this context, miR-142-5p was identified as a downstream binding target of CKMT2-AS1. Previous research has documented the dysregulation of miR-142-5p in various tumors.^22^^,^^23^ For instance, the application of miR-142-5p mimic in ovarian cancer cells significantly increased the viable cell counts of SKOV3.^24^ Conversely, in gastric cancer BG823 cells, where miR-142-5p was found to be downregulated, the introduction of miR-142-5p mimic led to a significant increase in apoptotic cells.^25^ These findings suggest that miR-142-5p may serve distinct roles across different tumor types. In the current investigation, a significant downregulation of miR-142-5p in HCC tissues and cell lines was observed, findings that resonate with prior reports concerning HBV-related HCC.^13^ Nevertheless, there remains a dearth of research addressing the regulatory interplay between CKMT2-AS1 and miR-142-5p in the context of HCC progression. Consequently, in vitro functional experiments were executed that elucidated the negative regulatory relationship between CKMT2-AS1 and miR-142-5p, indicating that this interaction may influence the biological behavior of HCC cells. However, it is worth noting that this investigation did not include in vivo evidence, which will undoubtedly be a critical focus for future research endeavors.

*IFITM3* plays a pivotal role in the regulation of various cellular biological processes,^26^ thereby significantly contributing to tumorigenesis and tumor progression. Recent investigations have demonstrated that *IFITM3* is highly expressed in HCC tissues, indicating a positive correlation between *IFITM3* levels and the risk of developing HCC.^27^ Furthermore, investigations at the tissue level have revealed a direct association between *IFITM3* mRNA expression and the malignancy of HCC. Notably, lower differentiation of HCC cells is linked to elevated levels of *IFITM3* mRNA. Additionally, patients with metastatic liver cancer exhibit higher *IFITM3* mRNA expression levels.^17^ Other studies have identified *IFITM3* as an oncogene in HCC.^28^ Mechanistically, some research suggests that *IFITM3* may modulate the Wnt/β-catenin signaling pathway and engage in the G0/G1 checkpoint, influencing the cell cycle during tumor development.^29^ Cellular experiments have shown that miR-142-5p is upregulated in HCC cells when treated with the miR-142-5p mimic, leading to a significant reduction in *IFITM3* mRNA levels. This finding confirms* IFITM3* as a target gene of miR-142-5p. However, this preliminary experimental study has primarily established the targeting relationship between miR-142-5p and *IFITM3*. This research primarily relied on an in vitro cell model, which may not accurately reflect the intricate in vivo conditions. The specific mechanisms underlying this interaction necessitate further investigation, and subsequent research should aim to validate these findings in appropriate animal models.

LncRNA CKMT2-AS1 was found to be significantly upregulated in HCC tissues and cell lines, while miR-142-5p exhibited a marked downregulation. The findings suggested that CKMT2-AS1 played a crucial role in promoting the progression of HCC. CKMT2-AS1 sponged miR-142-5p; thus, the downregulation of miR-142-5p effectively mitigates the inhibitory effects imposed by CKMT2-AS1 on cellular functions. Additionally, this research established a potential regulatory network involving lncRNA, miRNA, and mRNA, highlighting *IFITM3* as a pivotal target that might influence the cell cycle and behaviors of HCC cells. This study provides important insights into the regulatory mechanisms underlying the onset and progression of HCC, offering promising avenues for the identification of novel molecular markers and therapeutic targets for clinical applications in HCC management.

## Figures and Tables

**Supplementary Table 1.** Primers used in this study

Gene Name	Primer Sequence (5’-3’)	
lncRNA CKMT2-AS1	Forward	AACCTACCACTATAATCCA
	Reverse	ATTCTGTCCACTGTATCT
miR-142-5p	Forward	GCGCATAAGTAGAAAGC
	Reverse	AGTGCAGGGTCCGAGGTATT
*IFITM3*	Forward	GAGGACAGCCCCCAAACTAC
	Reverse	CTCCAGTCACATCACCCACC
Cel-miR-39	Forward	UCACCGGGUAAAUCAGCUUG
U6	Forward	ATTGGAACGATACAGAGAAGATT
	Reverse	GGAACGCTTCACGAATTTG
*GAPDH*	Forward	ACCACAGTCCATGCCATCAC
	Reverse	TCCACCACCCTGTTGCTGTA

## Data Availability

All data generated or analyzed during this study are included in this article. Further enquiries can be directed to the corresponding author.
